# Structural analysis and insertion study reveal the ideal sites for surface displaying foreign peptides on a betanodavirus-like particle

**DOI:** 10.1186/s13567-015-0294-9

**Published:** 2016-01-11

**Authors:** Junfeng Xie, Kunpeng Li, Yuanzhu Gao, Runqing Huang, Yuxiong Lai, Yan Shi, Shaowei Yang, Guohua Zhu, Qinfen Zhang, Jianguo He

**Affiliations:** State Key Laboratory of Biocontrol, MOE Key Laboratory of Aquatic Product Safety, Institute of Aquatic Economic Animals and Guangdong Province Key Laboratory for Aquatic Economic Animals, School of Life Sciences, Sun Yat-sen University, Guangzhou, 510275 China; Department of Nephrology, Guangdong General Hospital, Guangdong Academy of Medical Sciences, Guangzhou, 518080 China; School of Marine Sciences, Sun Yat-sen University, Guangzhou, 510275 China

## Abstract

Betanodavirus infection causes fatal disease of viral nervous necrosis in many cultured marine and freshwater fish worldwide and the virus-like particles (VLP) are effective vaccines against betanodavirus. But vaccine and viral vector designs of betanodavirus VLP based on their structures remain lacking. Here, the three-dimensional structure of orange-spotted grouper nervous necrosis virus (OGNNV) VLP (RBS) at 3.9 Å reveals the organization of capsid proteins (CP). Based on the structural results, seven putative important sites were selected to genetically insert a 6× histidine (His)-tag for VLP formation screen, resulting in four His-tagged VLP (HV) at positions N-terminus, Ala220, Pro292 and C-terminus. The His-tags of N-terminal HV (NHV) were concealed inside virions while those of 220HV and C-terminal HV (CHV) were displayed at the outer surface. NHV, 220HV and CHV maintained the same cell entry ability as RBS in the Asian sea bass (SB) cell line, indicating that their similar surface structures can be recognized by the cellular entry receptor(s). For application of vaccine design, chromatography-purified CHV could provoke NNV-specific antibody responses as strong as those of RBS in a sea bass immunization assay. Furthermore, in carrying capacity assays, N-terminus and Ala220 can only carry short peptides and C-terminus can even accommodate large protein such as GFP to generate fluorescent VLP (CGV). For application of a viral vector, CGV could be real-time visualized to enter SB cells in invasion study. All the results confirmed that the C-terminus of CP is a suitable site to accommodate foreign peptides for vaccine design and viral vector development.

**Electronic supplementary material:**

The online version of this article (doi:10.1186/s13567-015-0294-9) contains supplementary material, which is available to authorized users.

## Introduction

Piscine nodaviruses, the causal agents of viral nervous necrosis (VNN) or viral encephalopathy and retinopathy (VER), are members of the *BetanodavirusNodaviridae* family. Betanodaviruses are small, spherical, non-enveloped viruses with a bipartite single-stranded (+) RNA genome encapsulated by 180 molecules of a single self-assembly capsid protein (CP) [1]. They are important viruses in aquaculture because they can infect more than 40 marine fish species [2], especially high-value fish species, primarily at the larval and juvenile stages [3], which results in mass mortality and serious economic losses. To effectively control the disease, vaccine against betanodavirus is a pivotal strategy with general acceptance. Several types of vaccines have been reported, including poorly protective vaccines constituting recombinant CP [4, 5] or synthetic peptides derived from CP [6] as well as effective vaccines containing inactive betanodaviruses [7–9] or virus-like particles (VLP) [10–12]. VLP are well-structured particles with high similarity to native virions and they can assemble spontaneously from single (i.e. betanodavirus CP) or multiple structural proteins under specific conditions [13, 14]. VLP can be produced either eukaryotically or prokaryotically, and prokaryotic VLP production, in particular, presents advantages of high production, short duration, low cost, and easy manipulation. Theoretically, VLP are not infectious and they are safer than attenuated vaccines because of not containing viral genomes. Therefore, VLP is one of the most promising models for vaccine research and virology study.

Structural information of VLP is important in aiding vaccine design, modification or remodeling to develop the viral carriers (antigens or nucleic acids carrying vectors) with surface display techniques [15], to exploit multivalent vaccines [16], to manipulate the tropism of infection [17], and to achieve easy purification during vaccine production [18]. Cryo-Electron microscopy (cryo-EM) that allows visualization of macromolecules in their native state has been used to obtain the structural information of many virus particles. Combined with techniques of three-dimensional (3D) reconstruction, cryo-EM images of macromolecules can be used to study the high-resolution structure and interactions of macromolecules, helping to reveal the functions of assembly and invasion machineries of viral capsids. Several atomic resolution structures of virus (or VLP) and protein complexes have recently been reported [19, 20].

The OGNNV VLP was successfully produced prokaryotically and proved to be an effective vaccine against OGNNV [12]. In the present study, we want to verify the possibility of modification of betanodavirus VLP to achieve the purposes of vaccine research and viral vector development. Firstly, the 3D structure of native OGNNV VLP (RBS) at 3.9 Å resolution was determined. Not only the secondary structures but also some side-chain density were well defined and allowed us to identify the putative important sites for accommodating foreign peptides. Secondly, based on structural information, 6× histidine (His) tag and green fluorescent protein (GFP) were inserted into different positions by genetic approach to verify the importance of the predicted key sites by VLP production rate. The inward/outward displaying status and the carrying capacities of the sites of modified VLP were also verified. Finally, the potential applications of easily purified vaccines and viral carriers inserted at the C-terminal site were tested. Our results prove betanodavirus VLP can be modified by insertion and suggest the key sites for accommodating foreign peptides for the purpose of vaccine research and viral vector development.

## Materials and methods

### VLP production and purification

Full length *cp* gene of OGNNV (GenBank: AF534998) was cloned into modified pQE30 (Qiagen) and expressed prokaryotically in M15 *Escherichia coli* as described [12]. VLP were produced as follows. A volume of 80 mL seed culture grown overnight at 37 °C was inoculated equally into 8 flasks of 1 l LB-broth medium supplemented with 100 μg/mL ampicillin. When the cell density reached an OD600 of 0.3–0.4, the culture was cooled down to 30 °C and 0.9 mM IPTG was added for induction. After induction for 2 h at 30 °C, cells were harvested by centrifugation (5000 × *g*, 4 °C, 20 min), resuspended in 100 mL lysis buffer (pH 8.0 PBS with 1% Triton X-100 and 2 mM PMSF), lysed by sonication and centrifuged at 40 000 × *g* for 20 min. The whole cell pellets, supernatants and cell debris were saved for SDS-PAGE for a solubility test. Collected supernatant was ultracentrifuged at 250 000 ×* g* (Beckman Optima L-100XP) for 1 h against a 30% sucrose cushion. The pellet was resuspended in 4 mL of PBS (pH 8.0) and further purified using a 10–40% (w/w) sucrose gradient and ultracentrifuged at 250 000 × *g* for 3 h. Fractions of various buoyancies were collected and examined by SDS-PAGE and immunoblotting. The shape, size and integrity of the VLP were confirmed by the negative staining and transmission electron microscope (TEM) (JEOL JEM-1400) observation. The fractions containing fine structured and highly pure VLP were diluted with PBS and ultracentrifuged at 250 000 ×* g* for 1 h to remove the sucrose. The pellets were resuspended with PBS and the VLP (RBS or the modified VLP) were verified again by TEM. RBS was stored for the 3D structure research. Purified VLP were applied to SDS-PAGE and BCA Protein Assay Kit (Thermo) to determine the concentration and stored at −80 °C until use.

### Cryo-EM imaging, 3D reconstruction and model building

A 3.5 μL aliquot of the RBS sample was applied to a JG holey grid and blotted for 3 s in a chamber at 100% humidity and flash frozen using an FEI Vitrobot Mark IV. Particles were imaged with an FEI 300 kV Titan Krios cryo-electron microscope equipped with a Gatan Ultra-Scan4000 16-megapixel CCD at nominal magnification of 96 000×, corresponding to a final pixel size of 0.933 Å. The total dose was approximately 20 e^−^/Å^2^. The defocus values ranged from −1.0 to −3.0 µm.

Particles were picked up from the micrographs using the *e2boxer.py* program in *EMAN2* [21] and the contrast transfer function parameters were determined using *EMAN1.9* [22]. Article images (32 000) from 1359 frames were used in the final reconstruction. The data were randomly separated into two independent groups and *JSPR* was used to refine the center, orientation, defocus, astigmatism, and magnification of each particle and rebuild the final density map [23]. The resulting density maps were sharpened by applying the B-factor using the *proc3d* in *EMAN1.9* [22]. The resolution was assessed using the gold-standard by the 0.143 FSC criterion [24]. The local resolution was also calculated using the two independent reconstructions from halves of the total particles by *ResMap* [25].

All the protein subunit segmentation and visualizations were finished using UCSF *Chimera* [26]. The CP sequence was used to build a comparative model by *Phyre2* [27]. The model was then fitted into the density map using the ‘‘fit into map’’ module in UCSF *Chimera* [26] according to the previously reported methods [28]. Further refinement and optimization was done by the *Coot* [29] and *Phenix* [30]. Finally, we used the refined model instead of the initial comparative model to guide the segmentation of its corresponding density.

### Plasmid construction

Primers NHV-L and CP-R or RBS-L and CHV-R were used to amplify the *cp* gene from cDNA of RNA2 and the product was then inserted into pQE30 (Qiagen) using *Sac*I and *Hind*III or *EcoR*I and *Hind*III sites to generate pQE-His-CP or pQE-CP-His, respectively. These plasmids are used for expression of 6× His-tagged CP at N- or C-terminus. To generate the plasmids for expression of internal 6× His-tagged CP, overlap PCR was performed. The primers RBS-L and CP220HD/CP254HD/CP286HD/CP292HD/CP301HD or CP220HU/CP254HU/CP286HU/CP292HU/CP301HU and CPTAA-R were used to amplify the *cp* gene to generate upstream or downstream intermediate amplicons (IA). The corresponding upstream and downstream IA were purified and used as templates for amplifying by RBS-L and CPTAA-R to generate the final amplicons. The final amplicons were inserted into pQE30 using *EcoR*I and *Hind*III sites to generate five internal His-tag inserted plasmids, pQE-220HisCP, pQE-254HisCP, pQE-286HisCP, pQE-292HisCP, and pQE-301HisCP, for expression of internal 6 × His tagged VLP. The plasmid for expression of native VLP, namely RBS, is pQE-CP [12]. Overlap PCR was also performed to generate GFP-tagged CP. The primers EGFPNL/EGFPCL and EGFPNR/EGFPCR or NGV-L/CGV-L and CP-R/CGV-R were used to amplify *egfp* or *cp* gene to generate IA. The resulting IA were purified and used as template for amplifying by EGFPNL/CGV-L and CP-R/EGFPCR to generate the final amplicons of N-terminal or C-terminal tagged CP. For 220GFP-CP, the primers CGV-L/EGFPCL/220GV-L and 220GV-R/EGFPNR/CP-R were used to amplify three IA that were subsequently used as templates for two rounds of overlap PCR. All the final amplicons were inserted into pRSET-A (Invitrogen) using *Xba*I and *Hind*III sites. The resulting plasmids were named as pR-GFPCP, pR-220GFPCP and pR-CPGFP. All of these plasmids were transformed into M15 *E. coli* cells for expression. Sonication and gradient purification were performed to verify the production of different VLP as mentioned above. The primers used here are listed in Additional file 1.

### Immuno-EM

RBS or HV were applied onto carbon-filmed grids, left for 15 min, and blotted until nearly dry. Grids were then blocked by incubation face down on drops of 2% bovine serum albumin (BSA) for 30 min. Grids were incubated for 1 h with mouse anti-His monoclonal antibody (EarthOx, USA) at 100-fold dilution or mouse anti-VLP sera (polyclonal antibody produced in-house, total immunoglobulin) at 200-fold dilution. After that, grids were incubated for 1 h with goat anti-mouse IgG conjugated to 5 nm gold particles (Sigma) at 50-fold dilution. All incubations were performed at room temperature and grids were washed 3 times with 0.05% PBS-Tween 20 (vol/vol, PBST) between each step from the blocking step. VLP and gold particles were confirmed by negative staining and TEM observation.

### VLP invasion assay

The invasion assays were performed using RBS, HV and CGV on OGNNV-sensitive Asian sea bass (*Lates calcarifer*) fibroblast (SB) cells as previous described [12]. In brief, SB cells were grown on coverslip at 26 °C. VLP (0.3 μg/μL) diluted in minimal essential medium (Gibco) were incubated with SB cells at 0 °C for 1 h for attachment and invasion synchronization. After being thoroughly washed with PBS to remove excessive VLP, SB cells were received fresh medium and were switched to 26 °C to trigger invasion for 1.5 h. For observation of CGV in real-time, fluorescent microscopy was performed at the indicated time. For indirect immunofluorescence assays (IFA) [31], media were removed and cells were fixed with 4% paraformaldehyde for 30 min and then permeabilized for 5 min in 0.2% PBS-Triton X-100 (vol/vol) at room temperature. The cells were washed with 0.05% PBST (all wash steps below indicate three times of 0.05% PBST) and blocked with 5% FBS. IFA was performed at room temperature by incubating cells for 1 h with mouse anti-His monoclonal antibody (EarthOx, USA) at 500-fold dilution or mouse anti-VLP sera at 1000-fold dilution as primary antibodies. Cells were washed and treated with Alexa Fluor 488-labeled goat anti-mouse IgG antibody (Life technologies) at 1000-fold dilution as the secondary antibody for 1 h. Cells were washed and the cell nuclei were counterstained in red with PI (Sigma). Fluorescent microscopy was carried out using a Nikon Eclipse Ti fluorescent inverted microscope, and images were analyzed using Nikon NIS Elements imaging software (Br2 v3.21) to determine the location of virions.

### Affinity chromatography of HV

As described above for VLP production, cells expressing HV were induced, harvested, resuspended and sonicated in lysis buffer with addition of 10 mM imidazole. After centrifugation, a one bed volume of the collected supernatant was mixed with a half bed volume of nickel-nitrilotriacetic acid (Ni–NTA) matrices (Qiagen) that had been previously washed three times with lysis buffer for 2 h at 4 °C with rotation. The matrices were then washed six times with three bed volume of wash buffer (lysis buffer with addition of 26 mM imidazole) with 15 min rotation during each wash. Proteins bound to the matrices were eluted with a one bed volume of elution buffer (lysis buffer with addition of 250 mM imidazole) for 30 min at 4 °C with rotation. The eluted proteins were concentrated and the buffer was exchanged with PBS using Amicon^®^ Ultra Centrifugal Filters (Merck Millipore) according to the manufacturer’s instructions. Purified HV were subjected to SDS-PAGE and BCA protein assay kit (Thermo) for concentration determination, and to EM for structural completeness evaluation. Purified HV were stored at −80 °C until use.

### Sea bass immunization and antibody response detection

Healthy Asian sea bass juveniles with an average body weight of 5 g were maintained in flow-through freshwater at room temperature, aerated with an air pump and acclimated to the system for 3 days, at which time tissues were collected from 6 random fish for virus-free test and as control samples before injection (0 h). Feeding was suspended 12 h before vaccination and sampling. Sea bass were divided equally into ten groups with 40 fish per group. Two independent trials were conducted to determine the impact of immunization times (once and twice) to the vaccination efficiency and a 1-week interval was set before the booster. RBS or CHV were injected intramuscularly at doses of 15 μg/g fish body weight (FBW) as high concentration groups (V.H.: high concentration RBS; H.H.: high concentration CHV) or 1.5 μg/g FBW as low concentration groups (V.L. low concentration RBS, H.L. low concentration CHV) into the dorsum of anesthetized sea bass. PBS was also injected as negative controls. For antibody detection, thirty fish from each group in each trial were randomly selected. At indicated sampling time points (every week) post-immunization, fish sera were collected from the caudal vein and combined as one sample. Antibody titers of anti-VLP sera were determined by antigen-capture ELISA. All the experimental protocols concerning the handling of fish were in accordance with the requirements of the Institutional Animal Care and Use Committee of Sun Yat-sen University, P. R. China.

### Antigen-capture ELISA

Antigen-capture ELISA was performed as described [12] to determine the antibody titers of anti-VLP sera. In brief, 50 μL of diluted fish sera (1:100) were coated in a well of 96-well microtiter plate at 4 °C overnight. The plate was conducted to room temperature and washed three times with 200 μL PBS. After blocking with 5% BSA in PBS for 1 h and washing three times with 1% PBST (all wash steps below indicated three times of 200 μL 1% PBST), 1 ng VLP was added to each well and incubated for 1 h. After wash, the plate was incubated with in-house produced mouse anti-VLP antiserum at a dilution of 1:1000 in PBST for 1 h. The plate was washed and peroxidase-conjugated goat anti-mouse IgG (Sigma) at a dilution of 1:3000 was added and incubated for 1 h. Following thorough washes (five times), 100 μL of OPD substrate (Sigma) was added and the color development was conducted at room temperature. The reaction was stopped by the addition of 4 M sulfuric acid and the absorbance at 492 nm was determined. Statistical differences of titers from different groups were assessed by paired Student’s *t* tests. Numerical results are presented as mean ± standard deviation with 95% confidence intervals and *p* < 0.05 was considered statistically significant.

## Results

### The features of RBS capsid

**Figure 1:**
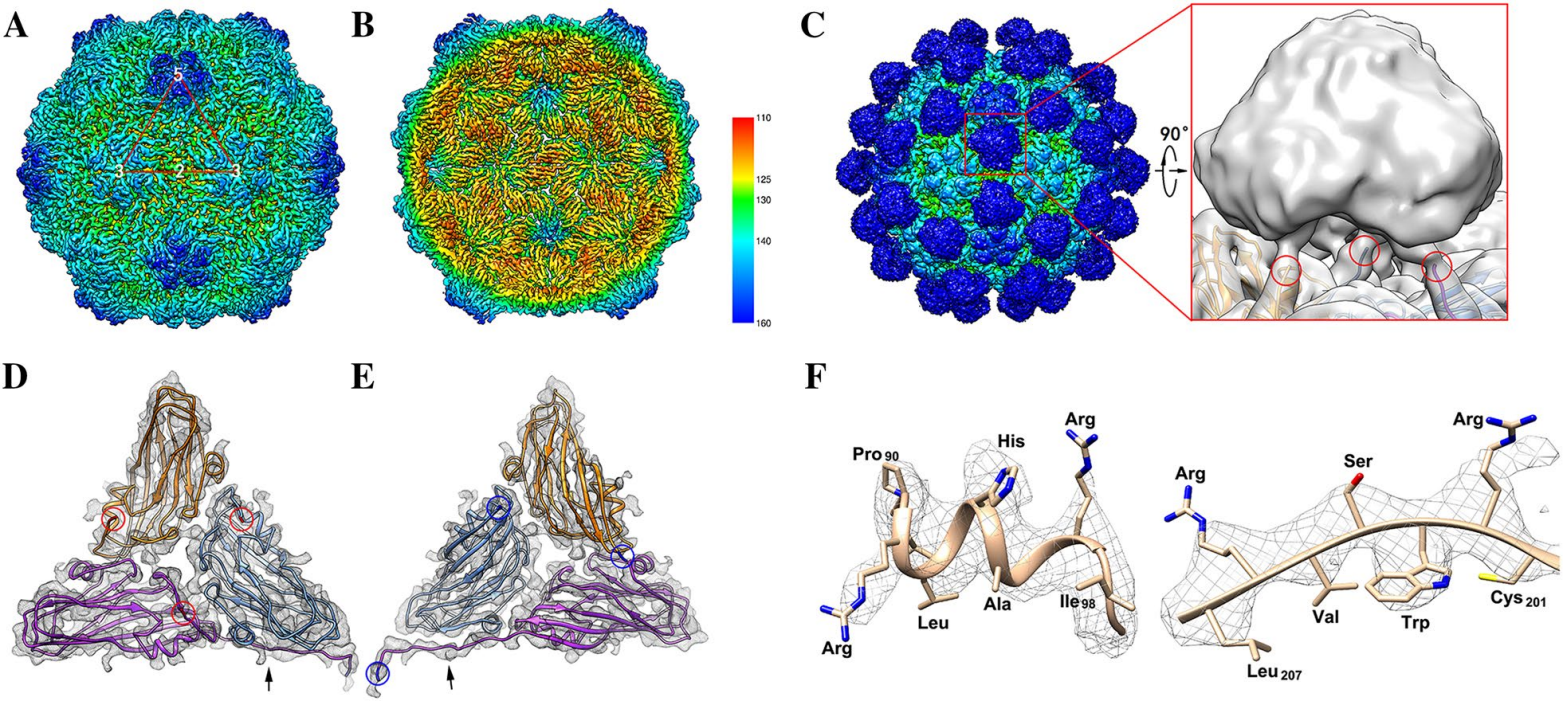
**Three dimensional structure of OGNNV VLP (RBS).** **A** The outside view of the RBS capsid. The red triangle demonstrates an asymmetry unit and the digits of 5, 3 and 2 show the position of the five, three and twofold axis. **B** The cut-way view shows the inward structure of the RBS capsid. The color bar shows the color scheme in the radius. The contour of **A** and **B** is 3.5. **C** The left one shows the surface map of RBS with the contour of 1.2. The color scheme is the same as **A** and **B**. It demonstrates that there are 60 protrusions outside the capsid shell, and the zoom in view (low passed to 8 Å) shows there are three linker loops that link the protrusion to the capsid. The red circles demonstrate the location of the residue Ala220. The contour of the right one is 0.6. **D**, **E** are the outside and inside view of an asymmetry unit density map superimposed by their models, respectively. The density maps are in gray, and the ribbon models of VPa, VPb and VPc are colored in golden, cornflower blue and purple, respectively. The outside view shows that the Ala220 residue (red circle) is at the surface of the capsid, while the inside view shows that all the residues of the N-terminal of the VPa and VPb (blue circles) are inside the capsid. For the VPc, 17 more residues which belong to the N domain form a loop (black arrow) across the VPb. **F** The features of a fragment of helix (left) and loop (right) at current resolution obviously demonstrate the big side chain densities. The models in golden color are superimposed in the density map (gray mesh). The contour of the map shown here is 3.2.

Final density map of the RBS capsid reconstructed by JSPR was estimated at 3.9 Å based on the “gold” standard FSC = 0.143 criterion (Additional file 2) (EMDB 6453). The 3D structure of RBS (Figures 1A and B) reveals that RBS has a capsid with 30 nm in diameter with T = 3 icosahedral symmetry (Additional file 3). Outside of the surface of the capsid, there are 60 protrusions located at the top of the center of each asymmetry unit, and with the protrusions, the diameter of the whole RBS enlarges to be around 38 nm (Figure 1C). There are three chemical identical subunits with slight conformational variations in the spatial location, VPa, VPb and VPc, in each asymmetric unit (Figures 1D and E) (also see Additional file 4). At current resolution, the alpha helix and beta sheets could be clearly identified, and most β-strands in the capsid were already separated (Figures 1D and E). Some distinctive side chains on helices, β-strands and loops could be identified (Figure 1F). Inside the capsid, there are some densities which belong to the randomly packaged RNA (Additional file 3). It was difficult to figure out the pattern of the RNA and the density became much weaker as the resolution was improved, because the reconstruction imposes icosahedral symmetry while the RNA should not be in the same symmetry.

A model for the capsid protein was built (PDB 3JBM) based on the density map at current resolution. Five VPa contribute to the densities around five-fold axis. VPb and VPc compose the densities of the hexagons together. We could only identify the densities of residues 52–220 of VPa and VPb. While for VPc, we could detect the densities of 17 more amino acid residues at the N-terminal (Figures 1D and E). These 17 amino acids compose a loop from the two fold axis and cross the VPb (Figure 1), then form a trident structure with another two N-terminus of the VPc at the three fold (Additional file 5).

Based on the density map and the model of the capsid, both N-terminals of the VPa, VPb and VPc are inside the capsid. If we low-pass the density map to 8 Å, three linker densities (residues 210–220) from each subunit of the asymmetry unit could be demonstrated connecting capsid shell to the protrusions at residues 220. There is no doubt that the protrusions densities belong to the residues 220–338, the C-terminus of the capsid protein. Accordingly, we separate CP into three independent domains: the N-terminal domain (N domain) (residues 1–50), the Shell domain (S domain) (residues 51–220) and the Protrusion domain (P domain) (221–338). The S domain is mainly composed of a canonical jelly-roll, and a β-barrel fold containing eight antiparallel β-strands. The S domain by itself forms a continuous capsid shell without any notable holes.

### The flexible protrusions and the putative sites for insertion study

Three P domains of VPa, b and c composed a trimeric protrusion in each asymmetry unit. Between S and P domains, there is a high flexible linker loop composed of residues 210–220 functioning as a connection (Figure 1C). The flexible linker loops are responsible for the spatial variation of the protrusions.

Our map demonstrates that the spatial variable protrusions outside the capsid are at relative low resolution. Local resolution analyses by *ResMap* show that the resolution of protrusion density is even worse than 10 Å (Additional file 6), resulting in no structural details of the protrusions being identified. Although protrusions have a low resolution, the structure we obtained allows identifying some residues for putative sites for VLP modification. Furthermore, the fine structure of capsid shell indicates that the insertion may influence the organization of VLP. Therefore, the modification study was focused on the protrusions and linker loops in order to display the foreign peptide. As a negative site for the virion surface displaying, the N-terminals are located inside the capsid shell. Consequently, residues 0 (N-terminus), 220, 254, 286, 292, 301, and 338 (C-terminus) of CP were selected as potential targets for subsequent insertion study.

### Modified VLP production

**Figure 2:**
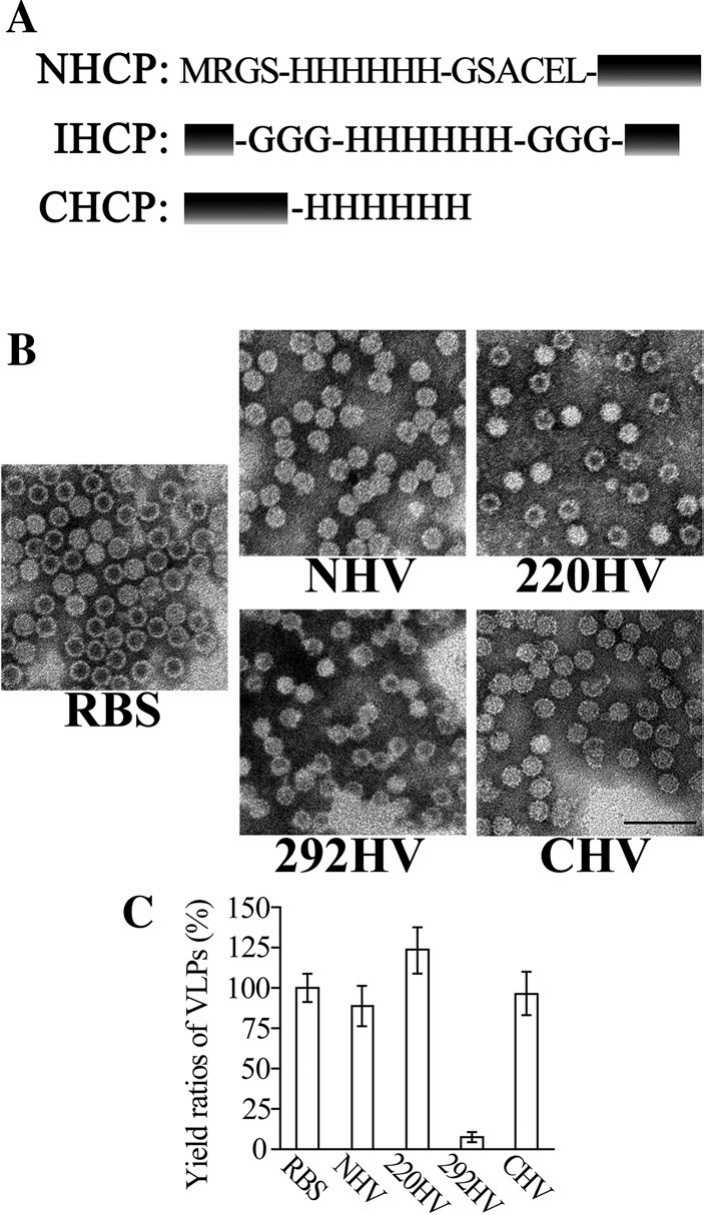
**Prokaryotic expression of HV** **. A** His-tag insertion design and the additional amino acid sequences. NHCP represents the N-terminal His-tagged CP, IHCP represents the internal His-tagged CP, and CHCP indicates the C-terminal His-tagged CP. The black blocks represent CP. **B** Electron microscopy of RBS and different HV. Magnification is 30 k and the bar indicates 100 nm. **C** Production ratio of different VLP in the same expression volume. The ratios were generated by normalization of production of HV to that of RBS. All the data represented means for at least 10 batches of VLP preparation with 2 standard deviations indicated by the error bars.

Based on the structure and model, we inserted a 6× His-tag into seven different positions of CP connected with flexible peptides as indicated in Figure 2A. All HCP were subjected to SDS-PAGE and immunoblotting to verify their expression and solubility after prokaryotic expression, sonication, and centrifugation (Figure 2). All of the HCP were correctly expressed after optimization except for 286HCP, which was expressed in very small quantities. However, the soluble forms of 292HCP and 301HCP were far less than those of other HCP (Additional file 7). Accordingly, the sequence of amino acid residues around positions Ala286, Pro292, and Trp301 were essential for CP expression and solubility. After sucrose gradient ultracentrifuge purification of the supernatants, we got tagged VLP of N-terminal HV (NHV), 220HV, 292HV, and C-terminal HV (CHV) (Figure 2B). Surprisingly, the highly expressed and soluble 254HCP is maintained as a monomer and cannot form VLP, suggesting that the position 254 of CP is important for VLP formation. Although different HV with shapes similar to that of RBS were produced, their quantities were remarkably different, as shown in Figure 2C. Comparing with the production of RBS (100%), the final quantities of NHV or CHV in the same culture volume were 89 ± 12.7% or 96 ± 13.3% while the amount of 220HV was 124 ± 13.2% and that of 292HV was less than 8% of RBS. 301HCP was unable to form sufficient amounts of particles (301HV, data not shown) for standard gradient ultracentrifugation, estimating that the production of 301HV was less than 1% of the RBS production. Furthermore, the broken particles were more frequently seen in 292HV and 301HV than in NHV, 220HV, and CHV. These results suggest that the internally inserted His-tag may influence the formation of highly structured particles while the insertions at N-/C-termini and linker loop were less affected. These results were consistent with the structural analysis, because the N-/C-termini and the linker loop are located far from the interaction domains and functional sites. Therefore, NHV, 220HV, and CHV were selected for further study because of their higher yields.

### Surface display of His-tag on HV verified by immuno-EM

**Figure 3:**
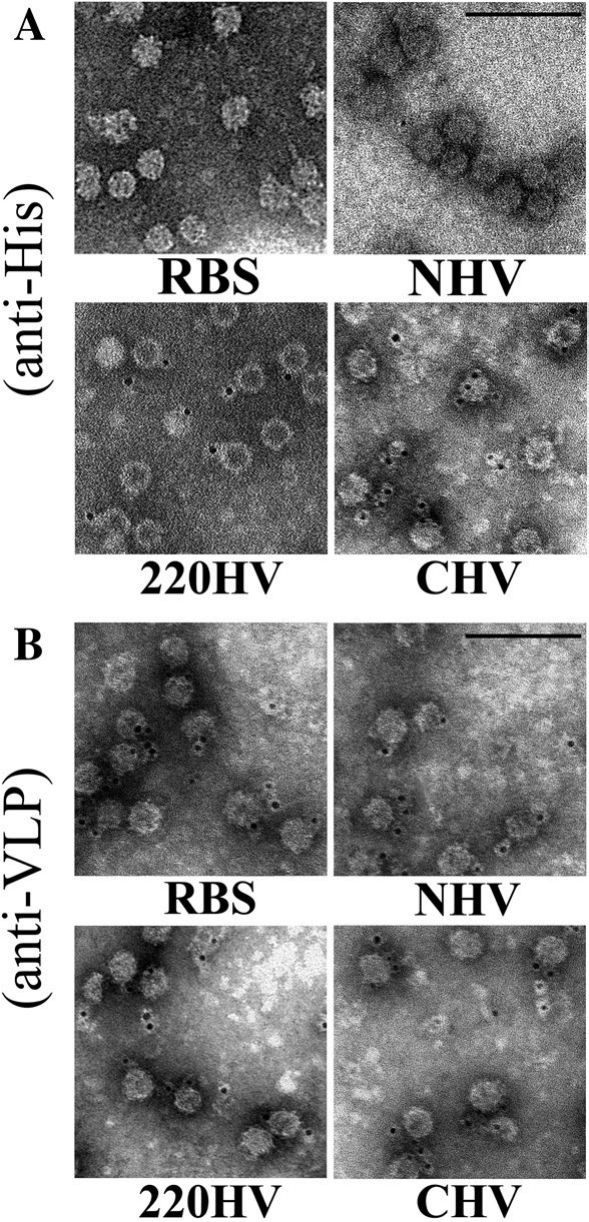
**Immuno-EM of different kinds of VLP.** RBS, NHV, 220HV, and CHV were immune-labeled with gold particles using mouse anti-his antibodies (**A**) or mouse anti-VLP sera (**B**) as the first antibody as indicated. The black dots are the 5 nm gold particles conjugated with goat anti-mouse secondary antibodies and the bars indicate 100 nm.

Immuno-EM assays of purified HV were performed to determine whether or not the His-tag presents at the surface of HV. The mouse anti-His antibodies and gold-labeled goat anti-mouse antibodies were used to detect surface displaying His-tags on fine structured VLP. As shown in Figure 3A, gold particles could be seen clearly around the 220HV and CHV but not be presented in the marginal places without VLP. This finding demonstrates that the His-tag is displayed on the surface of 220HV and CHV. NHV cannot be labeled with gold particles, which is similar to the negative control (RBS), suggesting that the N-terminal His-tag is not exposed outside of NHV. Structural information indicates that the trend of the N-terminal His-tag should be partially linked to the enclosed nucleic acid, which corresponds to the immune-EM results. By contrast, all types of VLP could be labeled with gold particles using polyclonal anti-VLP antisera (Figure 3B). These results indicate that the displayed His-tags do not affect the binding of anti-VLP antibodies.

### Cell entry ability of HV

**Figure 4:**
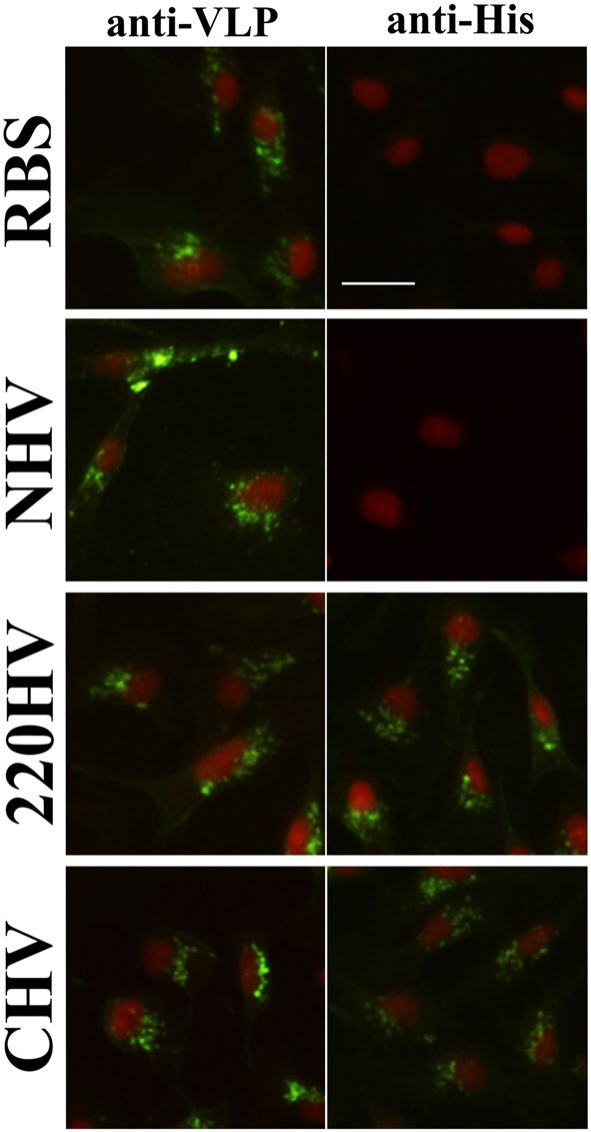
**HV maintained the cell entry ability.** The purified RBS and three types of HV were used to perform cell invasion assays. After 1.5 h incubation, the cells were fixed and IFA were performed using anti-His antibodies (left panels) and anti-VLP sera (right panels). The VLP in cells were labeled in green and the cell nuclei were counterstained in red. The His-tags of HV were stained positively when they were presented at the surface of the virions. The bar indicates 25 µm.

VLP invasion assays were performed to verify the entry process of HV and determine whether or not the displayed His-tags alter the surface structure of HV. Theoretically, the same entry process and comparative entry ability should be observed in the entry assays of HV and RBS if the surface structures of these particles are similar. In the assays, SB cells were incubated with HV, and RBS served as the positive control. The particles that had successfully entered were visualized by IFA. NHV, 220HV and CHV can enter SB cells via a pattern similar to that of RBS, as detected by IFA using anti-VLP antibody (left column of Figure 4). The particle entry was completed within 1.5 h and the particles aggregated next to or around the cell nuclei, indicating rapid endocytosis and they accumulate in some endosome-like compartments. These results demonstrate identical entry abilities between HV and RBS. Furthermore, the His-tags of 220HV and CHV could be detected by anti-His antibodies whereas the His-tag of NHV could not be labeled (right column of Figure 4). These findings were in accordance with the results of immune-EM. The invasion assays demonstrate that both native VLP (RBS) and modified VLP (HV) can specifically bind to the virus entry receptor(s) on the cell membrane and enter SB cells via the same endocytic pathway. Thus, the outer shell of HV is structurally indistinguishable from that of RBS or native virus, and the His-tags do not change the cell tropism of HV. By detecting the His-tags, HV and native VLP or viruses can be easily distinguished by immunoassay. Moreover, the key sites, such as Ala220 in the linker loop and C-terminus of CP, are excellent positions for accommodating foreign peptides on the outer surface of virions, exhibiting the potential application for viral vectors.

### Chromatographic purification of HV

**Figure 5:**
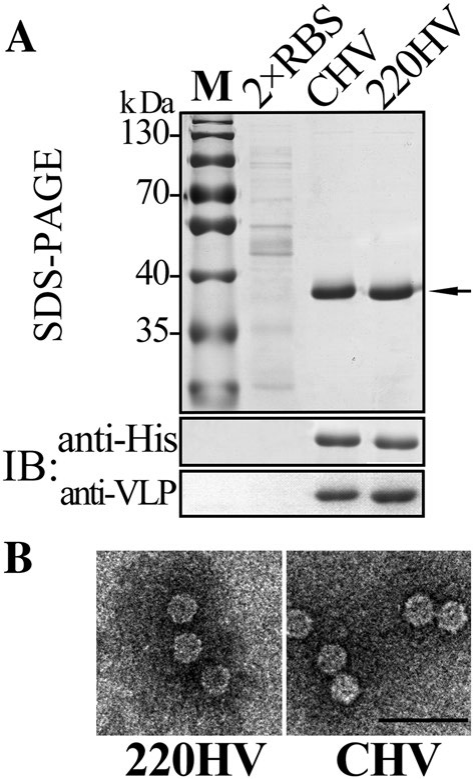
**Purification of HV by nickel-based affinity chromatography** **. A** SDS-PAGE and immunoblotting (IB) of purified HV and RBS. M represents protein marker and the HCP bands are indicated by an arrow. 2× RBS meant that the loading volume of RBS was two folds of those of other HV. **B** EM of chromatography-purified HV. The bar indicates 100 nm.

As an application of vaccine design for tagged VLP, we decided to test the purification function of His-tag by affinity chromatography because the tags are present on the surface of 220HV and CHV. Bacteria expressing RBS, 220HV, and CHV were harvested and broken by sonication. After centrifugation, the supernatants were purified by Ni-NTA beads and the purification results are shown in Figure 5. As an alternative method for VLP purification, affinity chromatography was used to purify 220HV and CHV, as detected by SDS-PAGE and immunoblotting (Figure 5A). Moreover, the purified products maintained the fine VLP structure, as revealed by EM (Figure 5B). Without the His-tag, RBS could not be enriched during purification. Moreover, the resulting quantities of 220HV and CHV produced from 1 L cell culture volume were 3.63 ± 0.55 and 3.84 ± 0.46 mg/L, respectively. Apparently, CHV was more effective than 220HV in affinity chromatography because the production quantities of 220HV were 29% more than those of CHV when using the ultracentrifugation method. This result suggests that the His-tags presented on the C-terminus are more successful than those displayed on Ala220 of the linker loop. The chromatography-purified HV can be used directly as vaccine although the concentration is lower than that obtained by ultracentrifugation. All the purification procedures were completed within 4 h, which means the present approach was much faster than the ultracentrifugation method. In a word, we successfully purified HV using a one-step affinity chromatography in a time- and effort- saving way.

### The immunogenicity of CHV

**Figure 6:**
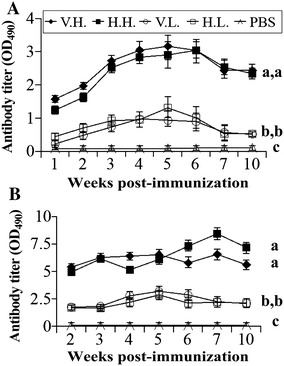
**NNV-specific antibody responses of sea bass juveniles.** RBS or CHV were used as vaccines in high concentration (V.H. or H.H.) and low concentration (V.L. or H.L.) to intramuscularly vaccinate sea bass juveniles, and PBS were used as the negative control. In each group, sera from 30 randomly selected fish were collected every week post-immunization for antigen-capture ELISA to monitor the antibody titers. NNV-specific antibody responses of sea bass juveniles stimulated by RBS or CHV with one (**A**) or two (**B**) injections are shown. The letters (a, b, and c) indicate the differentiation analysis results of each sample using the Student’s *t* test. The samples with the same letter contained no significant difference (*p* > 0.05) while the samples with different letters held significant difference (*p* < 0.05).

Chromatography-purified CHV was used as a vaccine to immunize sea bass juveniles to test the immunogenicity of CHV. The juveniles were vaccinated once or twice by intramuscular injection at doses of 15 μg/g FBW (H.H. group) and 1.5 μg/g FBW (H.L. group). RBS was used as the positive vaccine at the same concentrations (V.H. group and V.L. group) for comparison, and PBS served as the negative control. Fish sera from vaccinated and control sea bass were collected every week, and the NNV-specific antibody response was determined by antigen-capture ELISA. Figure 6A shows that both H.H. and V.H. stimulated levels of NNV-specific antibodies, being significantly higher than those of the PBS control (*p* < 0.01) when the fish were immunized once. The antibody response induced by CHV was nearly identical to that induced by RBS (*p* > 0.05), and the highest antibody titer was obtained at around 5 or 6 weeks post-immunization. Although H.L. or V.L. stimulated levels of antibody responses to a higher level than those induced by the PBS control (*p* < 0.01), their average antibody titers (0.81 U for H.L. or 0.66 U for V.L.) were threefold lower than those of H.H. (2.39 U) or V.H. (2.55 U). Therefore, immunization was performed twice to enhance the antibody response. As shown in Figure 6B, two injections of H.H. and V.H. stimulated very high levels of antibodies with average titers of 6.49 and 6.1 U, which were twofold higher than those obtained in H.H. or V.H. with one immunization. The high antibody titers induced by H.H. or V.H. remained stable for 10 weeks. Two injections of H.L. or V.L. improved the average antibody titers by two- to threefold compared with one immunization, although their titers were far lower than those obtained in H.H or V.H. samples. Nevertheless, the intensity of antibody response induced by two injections of H.L. was similar to that induced by H.H. with one injection. Therefore, the CHV vaccine purified by affinity chromatography was as efficient as RBS in stimulating high titers of specific antibodies in Asian sea bass at a dosage of 15 μg/g FBW (H.H.) with one injection.

### The carrying capacity of different insertion sites

**Figure 7:**
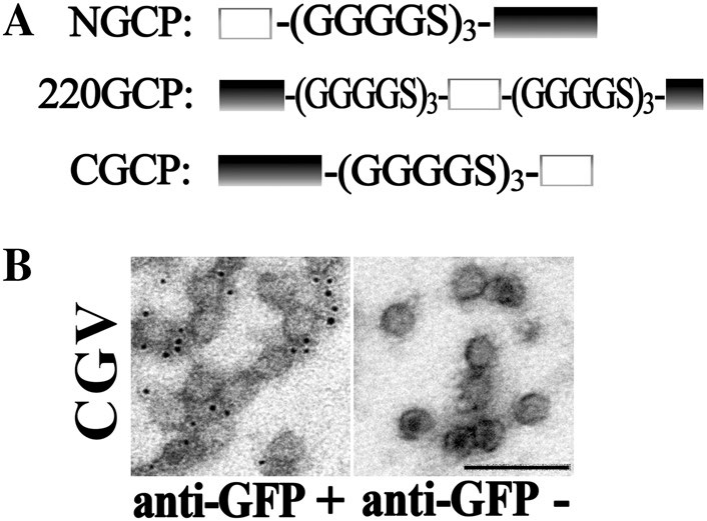
**GFP tagged VLP production and characterization** **. A** GFP insertion design and the additional amino acid sequences. NGCP represents the N-terminal GFP-tagged CP, 220GCP represents the Ala220 GFP-tagged CP, CGCP indicates the C-terminal GFP-tagged CP and (GGGGS)_3_ represents the flexible linker. The black blocks represent CP and the rectangle frames indicate GFP. **B** Immuno-EM was performed to detect the GFP display status of CGV and CGV was immune-labeled with 5 nm gold particles using mouse anti-GFP or not as first antibody as indicated. The bar indicates 100 nm.

Since the sites of N-/C-termini and Ala220 of linker loop are suitable positions for his-tag insertion, we went a step further to study their carrying capacity. NHV and 220HV can carry 16 and 12 amino acids, respectively, while CHV accommodates 6 amino acids, meaning that these three sites can carry short foreign peptides. To validate whether they can accommodate large functional proteins, GFP was inserted into these three sites. We found that no matter whether GFP fused directly with CP or connected to CP with a flexible linker, (GGGGS)_3_, the GFP-tagged CP on sites of N-terminus and Ala220 were unable to form VLP even when the fusion proteins were soluble. The same result was obtained when GFP fused directly to the CP C-terminus. However, when connected with the (GGGGS)_3_ linker, C-terminal GFP-tagged CP is soluble and can assemble into GFP-tagged VLP (CGV). The particle shape of CGV was similar to that of RBS (Figure 7B). CGV presents the GFP on the outer surface of particles as revealed by immune-EM (Figure 7B). Furthermore, the functional GFP can be visualized during the ultracentrifugation purification process. An additional file shows all the results on CGV production (Additional file 8). Therefore, the sites of N-terminus and Ala220 can only accommodate short peptides about 12–16 amino acids long while the C-terminus can carry functional proteins as large as GFP.

### Real-time observation of the entry process of CGV

**Figure 8:**
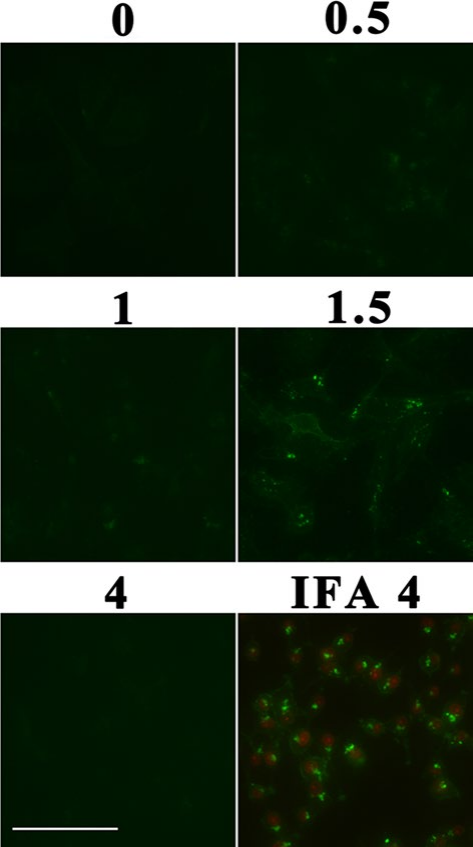
**Real-time observation of cell entry of CGV.** Purified CGV was used to invade SB cells and photographed under fluorescent microscopy at the time indicated. IFA shows that the panel resulted from IFA using anti-GFP as the first antibody. The bar indicates 100 µm.

Invasion assays of CGV were performed to find out whether CGV has the ability to invade culture cells, which means whether GFP can be carried into the cells via VLP as an application for viral vector. The entry process of CGV should be recorded in a real-time way by inverted fluorescent microscopy directly but not by indirect methods such as IFA. As shown in Figure 8, CGV not only can enter SB cells but can finish the entry process within 1.5 h, meaning that the entry speed and destination of CGV were similar to that of RBS. Because CGV lacks the cascade amplification of IFA, its overall fluorescent signals are weaker than Figure 4. Interestingly, the average fluorescent at 4 h post invasion (hpi) was weaker than those of 1–1.5 h. However, the GFP was detected with a strong fluorescent signal near the nuclei of anti-GFP antibody in IFA at this time point (Figure 8 IFA). These results probably suggest that the VLP structure of CGV was disassembled at 4 hpi but the GFP epitope was still detected by IFA. Consequently, CGV is suitable for viral in-cell traffic research, such as one-virus tracking or real-time virus entry study. Moreover, the C-terminus of CP is an excellent position for accommodating large functional proteins with outer surface displaying.

## Discussion

The accumulation of high-resolution viral structural information has facilitated the ability to modify VLP deliberately. Consequently, VLP can essentially function as molecular scaffolds for presenting foreign antigens by genetic insertion [32]. Both double- and single-stranded DNA and RNA viruses, including 14 families of viruses have been successfully generated by the chimeric VLP [33, 34]. Studies of chimeric VLP in betanodavirus are non-existent and the structural information is maintained at approximately 7.5 Å [35] and 23 Å resolution [36] according to the VLP of dragon grouper nervous necrosis virus and malabaricus grouper nervous necrosis virus. In this study, we report the 3D structures of OGNNV at 3.9 Å resolution determined by cryo-EM and single-particle reconstruction, and define the capsid protein containing N, S and P domains and a linker loop as the linkage between the S and P domains. Structures of the Flock House Virus (FHV) which belong to alphanodavirus have been studied in depth [37]. The crystal structure of nematode-infecting virus which is also related to the *Nodaviridae* was recently reported [38]. Compared with the reported structures of nodavirus, the canonical jelly-roll structure of the S domain is the most common. When Dali [39] is used to analyses the homologs of the S domain, the OGNNV S domain is best aligned to the nematode-infecting virus (Z score = 10.1) [38], while compared to that of FHV, the Z score is only 4.9. Interestingly, the S domain has a higher similarity to some plant virus shell proteins compared to that of the FHV which is thought to be the representative virus of *Alphanodavirus*. The Z score for the comparison to carnation mottle virus [40] and maize chlorotic mottle virus [41] is 8.0 and 5.6, respectively. In the reported alphanodaviruses, there is no obvious protrusion. The P domain of OGNNV composing a trimeric protrusion was similar to that of the nematode-infection virus. Although the reported betanodavirus structure is only in low resolution, it also showed that there are trimeric surface protrusions [36].

The high resolution structure of the capsid allows us to identify the structure details and the putative important sites for VLP modification. Based on the structural results, the N-terminals are located inside the capsid shell, and it can be a negative site for outer surface presentation. Ala220 is located at the tip of the capsid surface and it is located at the linker which connects the capsid shell and the protrusions together. Therefore, we should pay more attention to Ala220. Both C-terminus of the beta and gamma subunits are packaged inside the capsid shell in the mature alphanodavirus [42], while in the betanodavirus, all the C-termini of the CP stick out from the capsid shell. Although at the current state, we cannot tell the detailed locations of them in the protrusions, we can see that the protrusions are at the most outside of the RBS and all the C-termini are definitely packaged in the protrusions. Therefore, we can set N-/C-termini, Ala220 of the linker loop and several points among the protrusions as candidates that could be target sites for potential vaccine design. In the insertion study, different HCP were screened by VLP production, showing that the positions 0, 220, 292 and 338 can carry foreign peptides to assemble into VLP. Our results also show that the C-terminus and Ala220 (the linker loop) of CP are valuable sites for accommodating foreign peptides on the exterior surface, while the N-terminus accepts peptides in the interior of VLP, which is consistent with the structural results. Furthermore, the N-terminus and the linker loop can only carry short peptides while the C-terminus can accommodate large functional proteins such as GFP with a flexible linker. Cell invasion assays proved that NHV, 220HV, CHV and CGV maintain their cell tropism (entry receptor[s]) and entry ability (endocytosis pathway) as native VLP. Moreover, the application studies of tagged VLP show the potential use of C-terminal insertion. Addition of the His-tag to the C-terminal of CP does not alter the structure or immunogenicity of VLP, as proven by fish immunization studies, showing that the strategy of adding small tags to facilitate purification is feasible and practical in VLP vaccine development. It is noteworthy that equal dose of VLP induced relatively lower antibody titers in sea bass than in groupers [12], suggesting the lower VLP susceptibility of sea bass. CGV, the GFP carrying VLP, maintains the same entry ability as RBS and can be visualized in real-time for virus position tracking, revealing the potential application of viral vector development. To the best of our knowledge, this work is the first study to describe the exogenous-tagged VLP of betanodavirus.

Many studies have focused on the modification of the structure protein of either recombinant viruses or VLP to achieve basic structural and application research. For example, the core particle of the hepatitis B virus, namely HBc, has become one of the most popular carriers for the presentation of foreign peptides [43]. The foreign peptides are inserted either into the N-/C-termini of HBc or the immunodominant loop region located at the tip of the surface ‘‘spikes’’ on HBc particles by genetic fusion [44, 45], or linked to native HBc by chemical coupling [46], or attached to the fusion binding tag on HBc by protein–protein interaction [47]. FHV was also utilized as a display system to present the receptor von Willebrand A domain which can effectively bind the protective antigen of *Bacillus anthracis* at the surface of FHV VLP. The recombinant FHV VLP can neutralize the anthrax intoxication in vitro and in vivo, exhibiting the display potency of nodavirus VLP [48]. There is no modification study on epitope addition or chimeric VLP in betanodavirus, except for published reports about point mutation [49, 50] or terminal deletion [51]. We found that the C-terminus of OGNNV CP can accommodate the His-tag and functional GFP without changing the VLP structure, suggesting the possible application of the viral vector as developed in alphanodavirus.

Betanodaviruses infect many marine fish species [2], suggesting their strong infectivity to a wide range of hosts. OGNNV VLP can successfully enter several types of fish and mammalian cell lines determined by cell entry assays, probably via a clathrin-dependent endocytosis pathway (unpublished data). Thus, betanodavirus VLP is an easily delivered viral vector with the ability to enter different hosts, especially mammals. In respect of inducing the host’s immune response, several types of VLP have been proven to activate the innate immune system [52] and cellular immune system by pathogen-associated molecular pattern motifs [32]. VLP serve not only as scaffolds for presenting antigens derived from other pathogens in a suitable repetitive configuration but also as adjuvants to boost the immune response [34, 53]. The narrow host range (marine fish) of betanodavirus, in particular, may stand a good chance of activating the mammalian immune system quickly and vigorously because of limited contact between betanodaviruses and mammals. Consequently, betanodavirus VLP are perfect “self-adjuvanting” immunogen delivery platforms for foreign peptides. On the contrary, some researchers have added functional foreign peptides to change cell tropism [17] by reducing natural virus–cell interactions and conferring the ability to bind to new target receptors, resulting in a new strategy for developing novel delivery vehicles.

NHV, the VLP fused with His-tag on the N-terminus, is a useful type of viral vessel that differs from 220HV and CHV by its enclosed N-terminal His-tag. The key advantage of the N-terminal fusion vessel is that the fused short peptides can be protected by the VLP shield, which makes the peptides take effect in cells precisely when the VLP are lysed or disassembled without the influences of the VLP entry process. The His-tags of NHV were proved to be non-detectable by anti-His antibodies during immune-EM and IFA in cell entry assay when NHV presents as fine structural particles. Therefore, the structural completeness of NHV can be evaluated by anti-His antibodies against the His-tag marker. Thus, the timing and position of lytic virions can be revealed in a time-resolved manner. This strategy can provide more detailed information on intracellular traffic of betanodaviruses after endocytosis and NHV is a good tool for studying the betanodavirus endocytosis. A more sophisticated but similar strategy to elucidate the endocytosis details has been applied to HIV-1 studies. Time-resolved single-virus imaging and a virus population-based fusion assay were performed to delineate the cellular entry sites of HIV-1. The results revealed that complete HIV-1 fusion occurs in endosomal compartments but not at the plasma membrane of epithelial and lymphoid cells [54].

In summary, VLP of the betanodavirus, OGNNV, were studied structurally, the key sites for accommodating foreign peptides were investigated and the applications of easy purification and real-time viral tracking were performed, providing information for potential biotechnological applications.

## Additional files

10.1186/s13567-015-0294-9 **Primers used in this study.** The table shows all the primers used in the insertion study.
10.1186/s13567-015-0294-9 **The resolution of Cryo-EM structure of OGNNV VLP (RBS).** The resolution of the Cryo-EM density map is at 3.9 Å evaluated using gold-standard at FSC = 0.143.
10.1186/s13567-015-0294-9 **The averaged density distribution of the 3D reconstructions.** The mass densities of the RBS are spherically averaged and plotted as a function of the particle radius. Below a radius of 115 Å is the density of enclosed RNA fragments (The RNA fragments do not belong to the virus genome, they are arbitrarily enclosed bacterial RNA). The density distribution between 115–150 Å and 150–190 Å are the capsid and the protrusion respectively. In the capsid shell, each subunit arranged in a “jerry-roll” structure results in that the capsid shell looks like two layers (two density peaks).
10.1186/s13567-015-0294-9 **The side view of an asymmetry unit density map superimposed by the models.** The density maps are in gray, and the ribbon models of VPa, VPb and VPc are colored in the same scheme with that of Figures 1D and E. The outside view shows that the Ala220 residue (red circles) is at the surface of the capsid while the inside view shows that all the residues of the N-terminal of the S domain of VPa and VPb (blue circles) are inside the capsid.
10.1186/s13567-015-0294-9 **The N-terminal structure of the VPc.** The Cut-way view of the RBS is shown on left. The zoom in view on the right shows the loop (purple) of the N domain identified in VPc. Three VPc form a trident structure around the threefold axis.
10.1186/s13567-015-0294-9 **Local resolution analysis of RBS.** The local resolution analysis of RBS was performed using the program *ResMap*. The color bar shows the scheme in resolution. The results show that the inner capsid has the highest resolution while the loops, especially the loops at the surface, are in highest flexibility.
10.1186/s13567-015-0294-9 **Expression and solubility of different His-tagged CP. **Seven His-tagged CP were expressed in their optimal conditions. After bacteria cell collection, the samples of whole cells (W) were saved and the supernatants (S) and pellets (P) were collected respectively by centrifugation after sonication. M represents the protein marker and the “+” indicates the sample of purified CP. We can find the solubility of each HCP by evaluating the quantity of the CP band in S and P.
10.1186/s13567-015-0294-9 **Production and characterization of GFP-tagged VLP.** (A) SDS-PAGE of three GFP-tagged VLP. Three GFP-tagged CP were expressed in their optimal conditions. After bacteria cell collection, the samples of whole cells (W) were saved and the supernatants (S) and pellets (P) were collected respectively by centrifugation after sonication. M represents the protein marker and the arrow indicates the bands of fusion protein monomers. (B) Ultracentrifuge tubes in sucrose gradient purification of GFP-tagged VLP. The arrows indicate that the bands were clearly seen. They were collected, pelleted and resuspended with PBS as shown in (C). Light green fluorescent can be seen from the resuspended CGV but not from 220GV. The purified CGV and 220GV were observed by EM. Fine structured particles were only found in CGV sample (D). The bar indicates 100 nm.

## Abbreviations

OGNNV: Orange-spotted grouper nervous necrosis virus
CP: Capsid protein
VLP: Virus-like particle
3D: Three-dimensional
His: 6× histidine
HCP: His-tagged capsid protein
HV: His-tagged virus-like particle
NHV: N-terminal His-tagged virus-like particle
CHV: C-terminal His-tagged virus-like particle
CGV: C-terminal GFP-tagged virus-like particle
EM: Electron microscopy
RBS: OGNNV VLP
GFP: Green fluorescent protein
SB cell: Asian sea bass fibroblast cell
BSA: Bovine serum albumin
IFA: Indirect immunofluorescence assays
Ni–NTA: Nickel-nitrilotriacetic acid
FBW: Fish body weight
hpi: Hour post invasion
FHV: Flock house virus
