# Supplementary material for: Structural analysis and insertion study reveal the ideal sites for surface displaying foreign peptides on a betanodavirus-like particle
Source: Vet Res. 2016 Jan 11;47:16. doi: 10.1186/s13567-015-0294-9 (PMC4710043; doi:10.1186/s13567-015-0294-9)
Supplement: Supplementary file 1 — 10.1186/s13567-015-0294-9 Primers used in this study. The table shows all the primers used in the insertion study. [file 13567_2015_294_MOESM1_ESM.doc]

| Names | Sequence (5’ to 3’)^a^ | Purpose^b^ | |
| --- | --- | --- | --- |
| NHV-L | *CG*GAGCTC**atg**gtacgcaaaggtgagaag | Generate pQE-His-CP using  NHV-L and CP-R | |
| CP-R | *CCC*AAGCTT**tta**gttttccgagtcaaccctg |
| RBS-L | *CG*GAATTCATTAAAGAGGAGAAATTAACT**atg**gtacgcaaaggtgagaag | Generate pQE-CP-His using  RBS-L and CHV-R | |
| CHV-R | *CCC*AAGCTT**TCA**GTGGTGATGGTGGTGGTGgttttccgagtcaaccctgg |
| CP220HD | *CC*GTGGTGATGGTGGTGGTG*ACCACCTCC*agcggtggtctcttcagg | Generate IA 220F with RBS-L | Generate pQE-220HisCP using RBS-L, CP-R, 220F and 220R |
| CP220HU | *GT*CACCACCACCATCACCAC*GGTGGAGGT*cccatcatgacacaaggttc | Generate IA 220R with CP-R |
| CP254HD | *CC*GTGGTGATGGTGGTGGTG*ACCACCTCC*tccatcaggggcaatatcc | Generate IA 254F with RBS-L | Generate pQE-254HisCP using RBS-L, CP-R, 254F and 254R |
| CP254HU | *GT*CACCACCACCATCACCAC*GGTGGAGGT*gcagtcttccagctggacc | Generate IA 254R with CP-R |
| CP286HD | *CC*GTGGTGATGGTGGTGGTG*ACCACCTCC*agcaaacttcttgaggtgcc | Generate IA 286F with RBS-L | Generate pQE-286HisCP using RBS-L, CP-R, 286F and 286R |
| CP286HU | *GT*CACCACCACCATCACCAC*GGTGGAGGT*ggaaatgctggcacacct | Generate IA 286R with CP-R |
| CP292HD | *CC*GTGGTGATGGTGGTGGTG*ACCACCTCC*aggtgtgccagcatttcc | Generate IA 292F with RBS-L | Generate pQE-292HisCP using RBS-L, CP-R, 292F and 292R |
| CP292HU | *GT*CACCACCACCATCACCAC*GGTGGAGGT*gcaggctggtttcgctg | Generate IA 2292R with CP-R |
| CP301HD | *CC*GTGGTGATGGTGGTGGTG*ACCACCTCC*ccagatgccccagcg | Generate IA 301F with RBS-L | Generate pQE-301HisCP using RBS-L, CP-R, 301F and 301R |
| CP301HU | *GT*CACCACCACCATCACCAC*GGTGGAGGT*gacaacttcaacaagacgttcac | Generate IA 301R with CP-R |
| EGFPNL | *CG*TCTAGAAATAATTTTGTTTAACTTTAAGAAGGAGATATACAT**atg**ggcaaaggagaagaac | Generate IA N-EGFP-(GGGGS)_3_ with EGFP-N-R | Generate pR-GFPCP using EGFPNL, CP-R, N-EGFP-(GGGGS)_3_ and (GGGGS)_3_-CP |
| EGFPNR | GCTGCCGCCACCGCCGCTTCCGCCACCGCCGCTTCCACCGCCACCtttgtatagttcatccatgcc |
| NGV-L | GGTGGCGGTGGAAGCGGCGGTGGCGGAAGCGGCGGTGGCGGCAGCgtacgcaaaggtgagaag | Generate IA (GGGGS)_3_-CP With CP-R |
| CGV-L | *CG*TCTAGAAATAATTTTGTTTAACTTTAAGAAGGAGATATACAT**atg**gtacgcaaaggtgagaag | Generate IA CP-(GGGGS)_3_ with CGV-R | Generate pR-CPGFP using CGV-L, EGFPCR, CP-(GGGGS)_3_ and (GGGGS)_3_-EGFP |
| CGV-R | GCTGCCGCCACCGCCGCTTCCGCCACCGCCGCTTCCACCGCCACCgttttccgagtcaaccctg |
| EGFPCL | GGTGGCGGTGGAAGCGGCGGTGGCGGAAGCGGCGGTGGCGGCAGCggcaaaggagaagaac | Generate IA (GGGGS)_3_-EGFP with EGFPCR |
| EGFPCR | *CCC*aagctt**tta**tttgtatagttcatccatgcc |
| 220GV-L | GGTGGCGGTGGAAGCGGCGGTGGCGGAAGCGGCGGTGGCGGCAGCcccatcatgacacaaggtt | Generate IA (GGGGS)3-CCP with CP-R | |
| 220GV-R | GCTGCCGCCACCGCCGCTTCCGCCACCGCCGCTTCCACCGCCACCagcggtggtctcttcaggtgtc | Generate IA NCP-(GGGGS)_3_ with CGV-L | |
| *^c^* 1.Generate IA (GGGGS)_3_-EGFP-(GGGGS)_3_ using EGFPCL and EGFPCR  2.Generate IA N-(GGGGS)_3_-EGFP-(GGGGS)_3_ using CGV-L, EGFPCR, NCP-(GGGGS)_3_ and (GGGGS)_3_-EGFP-(GGGGS)_3_  3.Generate pR-220GFPCP using CGV-L, CP-R, N-(GGGGS)_3_-EGFP-(GGGGS)_3_ and (GGGGS)3-CCP | | | |

^a^ The lowercase letters indicate the sequence of *cp* or *gfp* gene, the underlined letters show the endonuclease sites, the circulated letters display the sequence of polyhistidine or (GGGGS)_3_, the italic letters indicate the additional sequences other than sequences mentioned above and the unlabeled uppercase letters show the sequence of ribosomal binding site.

^b^ The resulting plasmids are indicated. The inserts were generated by PCR or overlap PCR (generating pQE-220HisCP, pQE-286HisCP, pQE-292HisCP, pQE-301HisCP, pR-GFPCP, pR-220GFPCP and pR-CPGFP). The overlap PCR was performed using the corresponding intermediate amplicons (IA) as template with the indicated primers.

^c^ The process of generating pR-220GFPCP by series of overlap PCR.
