# Supplementary figures and images for: Structural analysis and insertion study reveal the ideal sites for surface displaying foreign peptides on a betanodavirus-like particle
Source: Vet Res. 2016 Jan 11;47:16. doi: 10.1186/s13567-015-0294-9 (PMC4710043; doi:10.1186/s13567-015-0294-9)

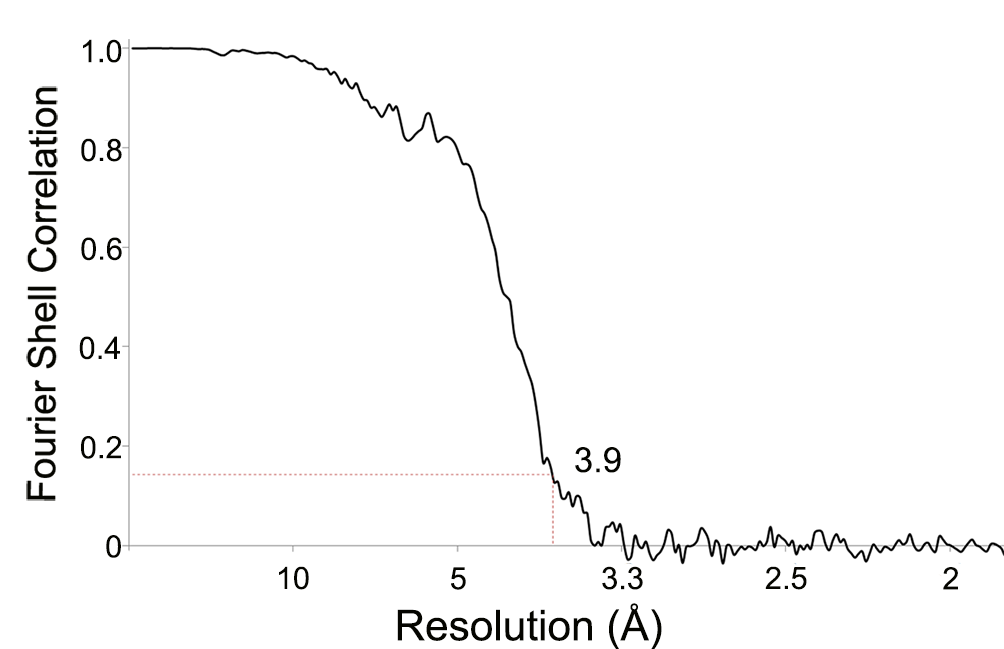

Supplement: Supplementary file 2 — 10.1186/s13567-015-0294-9 The resolution of Cryo-EM structure of OGNNV VLP (RBS). The resolution of the Cryo-EM density map is at 3.9 Å evaluated using gold-standard at FSC = 0.143. [file 13567_2015_294_MOESM2_ESM.tif]

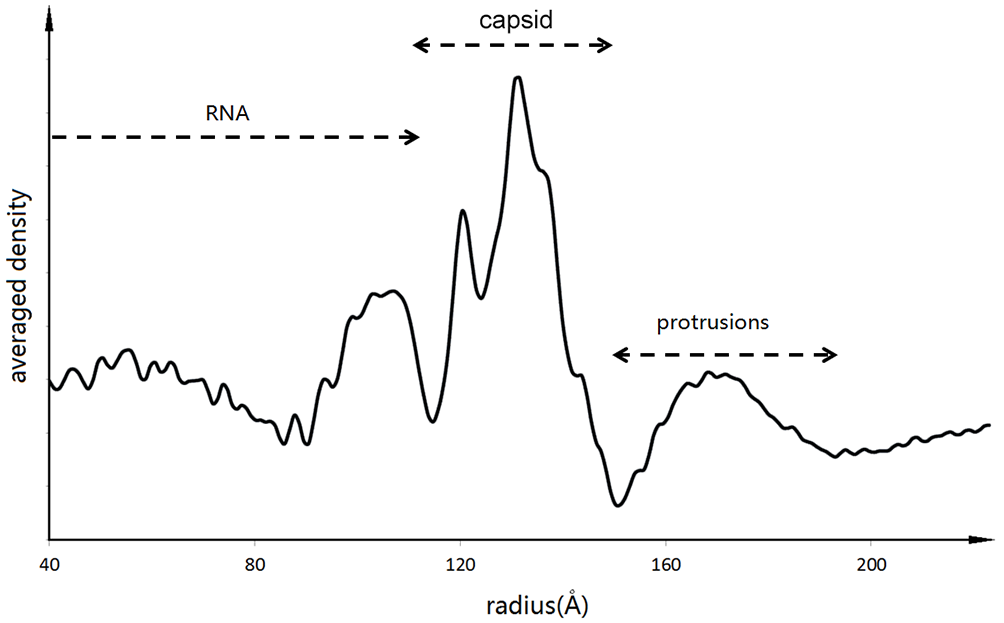

Supplement: Supplementary file 3 — 10.1186/s13567-015-0294-9 The averaged density distribution of the 3D reconstructions. The mass densities of the RBS are spherically averaged and plotted as a function of the particle radius. Below a radius of 115 Å is the density of enclosed RNA fragments (The RNA fragments do not belong to the virus genome, they are arbitrarily enclosed bacterial RNA). The density distribution between 115–150 Å and 150–190 Å are the capsid and the protrusion respectively. In the capsid shell, each subunit arranged in a “jerry-roll” structure results in that the capsid shell looks like two layers (two density peaks). [file 13567_2015_294_MOESM3_ESM.tif]

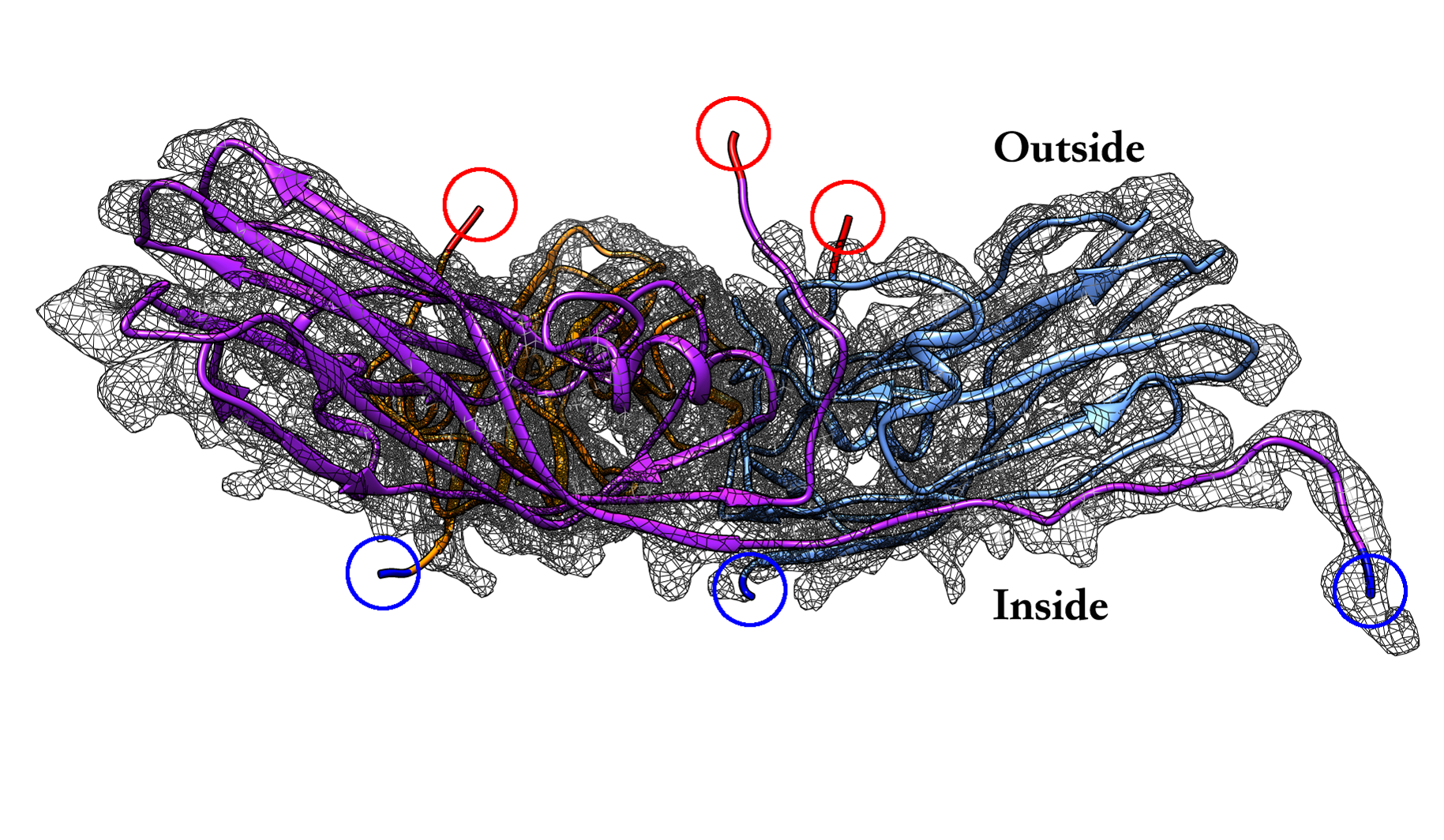

Supplement: Supplementary file 4 — 10.1186/s13567-015-0294-9 The side view of an asymmetry unit density map superimposed by the models. The density maps are in gray, and the ribbon models of VPa, VPb and VPc are colored in the same scheme with that of Figures 1D and E. The outside view shows that the Ala220 residue (red circles) is at the surface of the capsid while the inside view shows that all the residues of the N-terminal of the S domain of VPa and VPb (blue circles) are inside the capsid. [file 13567_2015_294_MOESM4_ESM.tif]

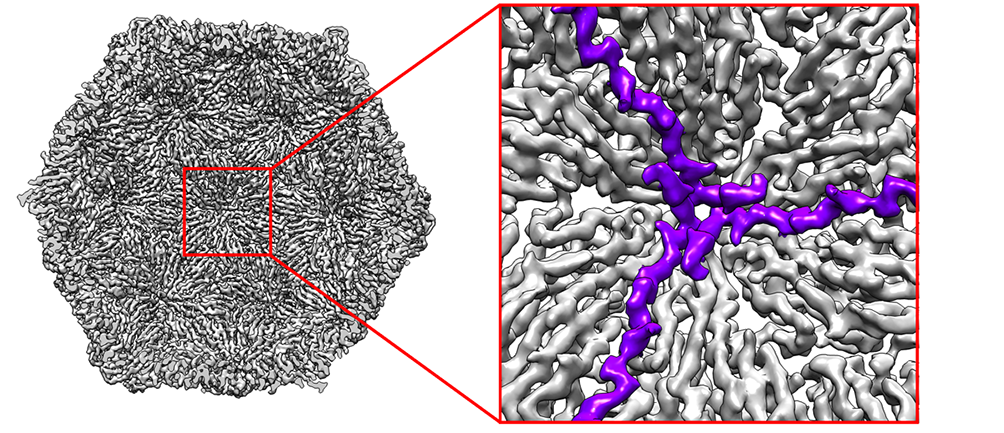

Supplement: Supplementary file 5 — 10.1186/s13567-015-0294-9 The N-terminal structure of the VPc. The Cut-way view of the RBS is shown on left. The zoom in view on the right shows the loop (purple) of the N domain identified in VPc. Three VPc form a trident structure around the threefold axis. [file 13567_2015_294_MOESM5_ESM.tif]

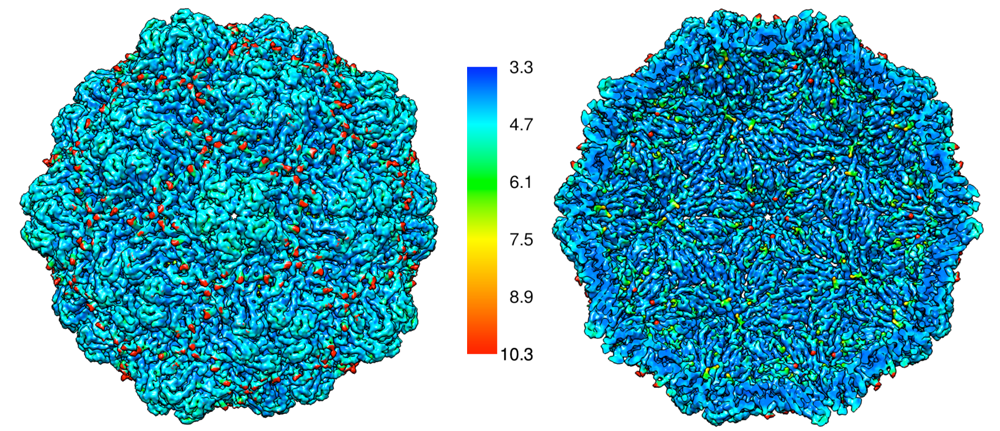

Supplement: Supplementary file 6 — 10.1186/s13567-015-0294-9 Local resolution analysis of RBS. The local resolution analysis of RBS was performed using the program ResMap. The color bar shows the scheme in resolution. The results show that the inner capsid has the highest resolution while the loops, especially the loops at the surface, are in highest flexibility. [file 13567_2015_294_MOESM6_ESM.tif]

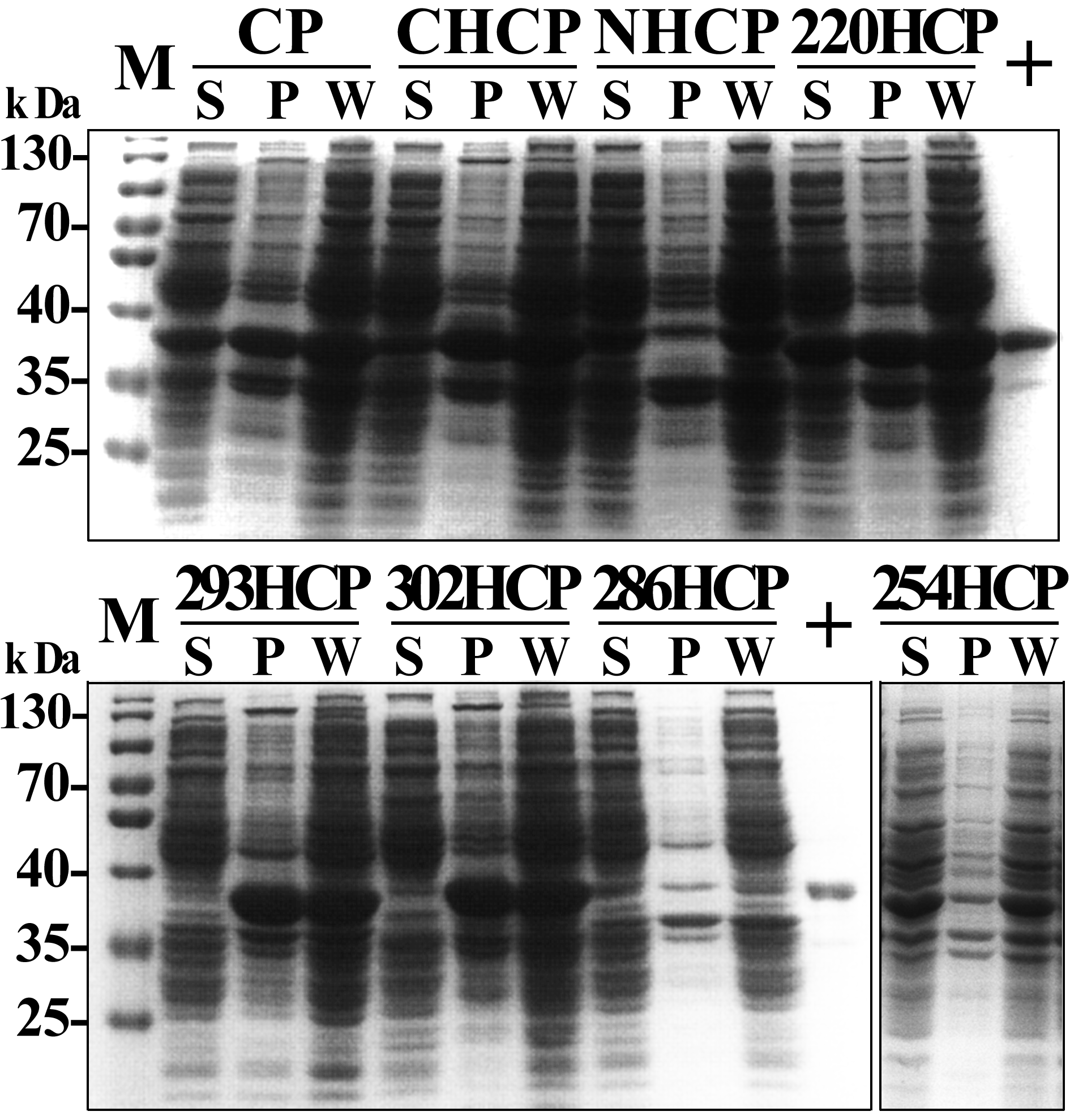

Supplement: Supplementary file 7 — 10.1186/s13567-015-0294-9 Expression and solubility of different His-tagged CP. Seven His-tagged CP were expressed in their optimal conditions. After bacteria cell collection, the samples of whole cells (W) were saved and the supernatants (S) and pellets (P) were collected respectively by centrifugation after sonication. M represents the protein marker and the “+” indicates the sample of purified CP. We can find the solubility of each HCP by evaluating the quantity of the CP band in S and P. [file 13567_2015_294_MOESM7_ESM.tif]

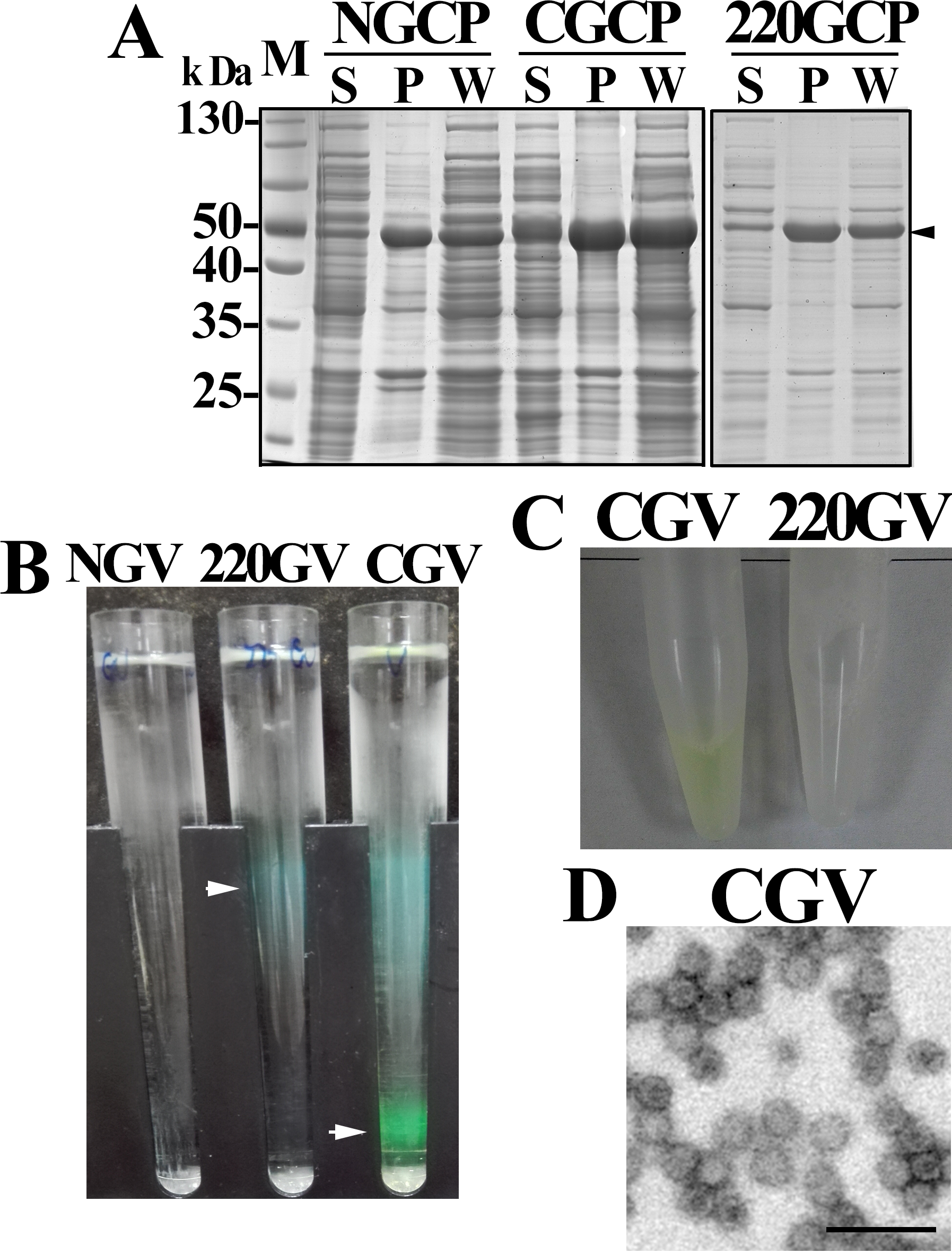

Supplement: Supplementary file 8 — 10.1186/s13567-015-0294-9 Production and characterization of GFP-tagged VLP. (A) SDS-PAGE of three GFP-tagged VLP. Three GFP-tagged CP were expressed in their optimal conditions. After bacteria cell collection, the samples of whole cells (W) were saved and the supernatants (S) and pellets (P) were collected respectively by centrifugation after sonication. M represents the protein marker and the arrow indicates the bands of fusion protein monomers. (B) Ultracentrifuge tubes in sucrose gradient purification of GFP-tagged VLP. The arrows indicate that the bands were clearly seen. They were collected, pelleted and resuspended with PBS as shown in (C). Light green fluorescent can be seen from the resuspended CGV but not from 220GV. The purified CGV and 220GV were observed by EM. Fine structured particles were only found in CGV sample (D). The bar indicates 100 nm. [file 13567_2015_294_MOESM8_ESM.tif]
